# TRIO RVEMVS: A Bayesian framework for rare variant association analysis with expectation-maximization variable selection using family trio data

**DOI:** 10.1371/journal.pone.0314502

**Published:** 2024-12-04

**Authors:** Duo Yu, Matthew Koslovsky, Margaret C. Steiner, Kusha Mohammadi, Chenguang Zhang, Michael D. Swartz

**Affiliations:** 1 Division of Biostatistics, Data Science Institute, Medical College of Wisconsin, Milwaukee, Wisconsin, United States of America; 2 Department of Statistics, Colorado State University, Fort Collins, Colorado, United States of America; 3 Department of Human Genetics, University of Chicago, Chicago, Illinois, United States of America; 4 Department of Biostatistics and Data Management, Regeneron Pharmaceuticals, Inc., Tarrytown, New York, United States of America; 5 Biostatistics and Research Decision Sciences, Merck & Co., Inc., North Wales, Pennsylvania, United States of America; 6 Department of Biostatistics and Data Science, School of Public Health, The University of Texas Health Science Center at Houston, Houston, Texas, United States of America; Rutgers The State University of New Jersey, UNITED STATES OF AMERICA

## Abstract

It is commonly reported that rare variants may be more functionally related to complex diseases than common variants. However, individual rare variant association tests remain challenging due to low minor allele frequency in the available samples. This paper proposes an expectation maximization variable selection (EMVS) method to simultaneously detect common and rare variants at the individual variant level using family trio data. TRIO_RVEMVS was assessed in both large (1500 families) and small (350 families) datasets based on simulation. The performance of TRIO_RVEMVS was compared with gene-level kernel and burden association tests that use pedigree data (PedGene) and rare-variant extensions of the transmission disequilibrium test (RV-TDT). At the region level, TRIO_RVEMVS outperformed PedGene and RV-TDT when common variants were included. TRIO_RVEMVS performed competitively with PedGene and outperformed RV-TDT when the analysis was only restricted to rare variants. At the individual variants level, with 1,500 trios, the average true positive rate of individual rare variants that were polymorphic across 500 datasets was 12.20%, and the average false positive rate was 0.74%. In the datasets with 350 trios, the average true and false positive rates of individual rare variants were 13.10% and 1.30%, respectively. When applying TRIO_RVEMVS to real data from the Gabriella Miller Kids First Pediatric Research Program, it identified 3 rare variants in q24.21 and q24.22 associated with the risk of orofacial clefts in the Kids First European population.

## Introduction

Birth defects are prevalent, occurring in 1 out of every 33 babies born annually in the United States, and are the primary cause of infant mortality, responsible for 20% of all infant deaths [1]. The impact of birth defects may be underestimated in mortality statistics [2], thus understanding the etiology of these major birth defects remains a research priority in birth defects epidemiology. Family data supports the hypothesis that a significant component of the risk for various birth defects stems from genetic variation [3–6]. Recent genome-wide association studies have identified common SNPs associated with varying birth defects, including obstructive heart defects (OHDs) [7], multiple congenital heart defect (CHD) phenotypes [8], conotruncal heart defects (CTD) [9], left-sided lesions (LSL) [10], and tetralogy of Fallot [11]. However, the identified variants only account for a small portion of the heritability. Part of the missing genetic heritability is thought to reside in rare variants, which are largely undetectable through genome-wide association platforms [12–17].

Although next-generation sequencing allows researchers to sequence each variant along the genome, there are statistical challenges inherent in identifying which rare variants are associated with disease. The power of traditional association methods for SNPs depends on allele frequency; the low frequency of minor alleles may reduce the power to analyze rare variants [18–22]. Previous methods are based on global tests that pool rare variants in a region to test the association with diseases. These global tests can be classified into burden tests (such as Cohort Allelic Sums Test (CAST) [23], the Collapsed Multivariate Collapsing Method (CMC) [24], and the Variable Threshold (VT) method [25, 26])), or quadratic tests (like C-alpha test [27] and the Sequence Kernel Association Test (SKAT) [28]).

Some methods have been developed that can identify specific rare variants that drive association within a given region of interest, as well as include common variants [24, 29–31]. However, these methods are based on a case-control design and are therefore subject to bias from population stratification [31–34]. Diseases that affect young children, like birth defects, are good candidates for family-based study designs, such as parent-child trios, which allow for more robust methods to analyze genetic data, including both common and rare variants, in the presence of population substructure [32, 35, 36]. Methods that take advantage of pedigree data for the genetic variant association, such as PedGene [37], and RV-TDT [38] have been developed. PedGene extends kernel and burden statistics for unrelated case-control data to include known pedigree relationships, which can account for the population-structured data [37]. Similarly, to avoid the spurious associations derived from the population-based method when the population substructure and admixture exist, RV-TDT extends commonly used population-based methods to analyze the association of rare variants in population-structured data, including aforementioned CMC [24] and VT [25]. However, none of these methods can detect individual rare variants within the region of interest. In this study, we proposed TRIO_RVEMVS, a Bayesian framework for individual rare variant association analysis with expectation-maximization variable selection using family trio data, which can simultaneously detect common and rare variants at the individual variant level.

The paper is organized as follows: We begin by constructing the likelihood of common and rare variants using case-parent trios. Next, we detail the Bayesian framework of TRIO_RVEMVS, which includes specifying priors for common and rare variants’ coefficients, conducting posterior inference using the EM algorithm, and tuning selection parameters. To assess TRIO_RVEMVS, we perform simulations on both large (1500 case-trios) and small (350 case-trios) datasets and compare the results with those obtained using PedGene and RV-TDT. We then apply TRIO_RVEMVS to a real-world trio dataset from the Gabriella Miller Kids First Pediatric Research Program consisting of trios with a child suffering from cleft lip with or without cleft palate (CL+P). The paper concludes with a discussion of our findings.

## Constructing the likelihood of common and rare variants using case-parents trios

In the case-parent trio design, small nuclear families are collected, where the child is affected by the disease or phenotype of interest. Then, the affected child and both parents are genotyped. Assuming each family can be phased, we denote the haplotype pair of the child [39]

$$g=(g_{m},g_{f})$$

where *g*_*m*_ and *g*_*f*_ denote the haplotypes inherited from the mother and father, respectively. Let *D*^+^ represent the child is diseased, Θ denote the transmission parameters, and denote the parental haplotype pairs from mother and father as *G*_*m*_ and *G*_*f*_, respectively. To model the sampling distribution of the case-trio family data, we propose to use the conditional logistic regression likelihood to model the probability of haplotype transmission from parents to the diseased child, which is motivated by the literature [39–41]. In more detail, the sampling distribution for observing a case trio can first be expressed as

$$P\left(g,G_{m},G_{f}|\Theta ,D^{+}\right)=P\left(g|G_{m},G_{f},\Theta ,D^{+}\right)P\left(G_{m},G_{f}|\Theta ,D^{+}\right),$$

Due to Mendelian laws of inheritance, we can assume the transmission parameters contained in Θ are conditionally independent of the parents’ genotypes *G*_*m*_ and *G*_*f*_, given that the child is diseased. Since the trios are sampled through the diseased child, there is no information regarding Θ in the sampling distribution of the parent’s haplotypes, which implies

$$P\left(G_{m},G_{f}|\Theta ,D^{+}\right)=P\left(G_{m},G_{f}|D^{+}\right),$$

and

$$\begin{array}{c} P\left(g,G_{m},G_{f}|\Theta ,D^{+}\right)\propto P\left(g|G_{m},G_{f},\Theta ,D^{+}\right). \end{array}$$ (1)

Eq (1) implies the sampling distribution will based on *P*(*g*|*G*_*m*_, *G*_*f*_, Θ, *D*^+^), which can be generally modeled by a conditional logistic regression according to previous studies [39–41].

The conditional probability of disease given the haplotypes of parents can be derived similarly as in [40],

$$\begin{array}{c} P\left(g|G_{m},G_{f},\Theta ,D^{+}\right)=\frac{P(D^{+}|g,\Theta )}{\sum_{j=1}^{4}P\left(D^{+}|g_{j},\Theta \right)}, \end{array}$$ (2)

where *g*_*j*_ denotes one of the four different haplotype pairs inheritable from parents, *j* = 1, 2, 3, 4. We use a logistic regression modeling framework to include both common and rare variants in the sampling distribution as

$$\begin{array}{c} \text{log}\left\{\frac{P(D^{+}|g,\Theta )}{1-P(D^{+}|g,\Theta )}\right\}=g_{c}\beta +g_{r}\alpha , \end{array}$$ (3)

where *g*_*c*_ is a 1 × *S* vector which denotes the common SNPs, *β* is a *S* × 1 coefficient vector, *g*_*r*_ is a 1 × *L* vector representing the rare SNPs, and *α* represents the effect of the selected rare variants on risk. For rare diseases, we can assume 1 − *P*(*D*^+^|*g*, Θ) ≃ 1. Therefore, Eq (3) simplifies to

$$\begin{array}{c} \text{log}\;P\left(D^{+}|g,\Theta \right)=g_{c}\beta +g_{r}\alpha , \end{array}$$ (4)

Similarly,

$$\begin{array}{c} \text{log}\;P\left(D^{+}|g_{j},\Theta \right)=g_{cj}\beta +g_{rj}\alpha , \end{array}$$ (5)

where *g*_*cj*_ and *g*_*rj*_ are the common and rare variants of *g*_*j*_. With Eqs (4) and (5), finally, Eq (2) can be expressed as

$$\begin{array}{c} P\left(g|G_{m},G_{f},\Theta ,D^{+}\right)=\frac{exp(g_{c}\beta +g_{r}\alpha )}{\sum_{j=1}^{4}\;exp\left(g_{cj}\beta +g_{rj}\alpha \right)}. \end{array}$$ (6)

Eq (6) can be understood as the likelihood for a 1:3 matched case-control design, where the affected child plays the role as the case and is matched with the 3 other possible genetic configurations of children that could have been offspring from the same parents. These other configurations are commonly called pseudo-siblings. Therefore, the conditional likelihood function for trios is given by

$$\begin{array}{c} L\left(g|G_{m},G_{f},\Theta ,D^{+}\right)=\prod_{n=1}^{N}P\left(g_{n}|G_{nm},G_{nf},D^{+},\Theta \right), \end{array}$$ (7)

where **g** denotes the collection of haplotype pairs of children from the case-trio data, **G**_**m**_ and **G**_*f*_ are the collections of haplotype pairs of parents in the case-trio data, *n* = 1, 2, ⋯, *N* are the indexes of families, and *P*(*g*_*n*_|*G*_*nm*_, *G*_*nf*_, *D*^+^, Θ) can be calculated through Eq (6).

## Trio rare variants EMVS (TRIO_RVEMVS)

The sampling distribution and likelihood for trio data using common variants have been previously discussed [35, 39–42], modeling the probability of transmitting genes to the affected child. In the previous section, we show the probability of transmission can be extended to incorporate rare variants and be written as Eq (6). In summary, trio data are modeled using conditional logistic regression, similar to the model for 1:3 matched case-control data. Here, the affected child is matched with the 3 “pseudo-siblings” who could potentially be offspring of the parents. Using this sampling distribution as our likelihood, we proceed to outline the remaining mathematical details to develop a Bayesian framework for the variable selection for both common and rare variants associated with disease and implement the EM algorithm to compute the posterior quantities of interest [43].

### Hierarchical priors

In this section, we construct the prior distributions to model inclusion in the risk model for each common and rare variant. For common variant selection, we used a single binary indicator, *γ*_*s*_, to denote whether a common variant is included in the model. For rare variant selection, we implemented a dual selection indicator structure, which was first used in genetics for common variants with multiple alleles [42] and later modified for use in a Bayesian sparse group selection framework [44]. Specifically, we used a binary indicator, *η*_*r*_, to indicate a group of related rare variants (typically those within the same gene) and a second indicator, λ_*rj*_, to indicate individual variants within group *r*. Each selection indicator follows a Bernoulli distribution, with a prior inclusion probability parameter *π*_*i*_ (*i* = 1, 2, 3):

$$\begin{array}{c} \begin{array}{cc} & \gamma_{s}|\pi_{1}∼\text{Ber}\left(\pi_{1}\right),\;s=1,2,\cdots ,S,\\ & \eta_{r}|\pi_{2}∼\text{Ber}\left(\pi_{2}\right),\;r=1,2,\cdots ,R,\\ & p\left(\lambda_{rj}|\eta_{r},\pi_{3}\right)=\eta_{r}\pi_{3}^{\lambda_{rj}}{\left(1-\pi_{3}\right)}^{1-\lambda_{rj}}+\left(1-\eta_{r}\right)\delta_{0},\;j=1,2,\cdots ,J_{r}, \end{array} \end{array}$$ (8)

where *δ*_0_ is point mass at zero, *S* denotes the total number of common variants, *R* denote the total number of regions, and *J*_*r*_ denotes the total number of rare variants in region *r*. If *γ*_*s*_ = 1, it indicates that a common variant is included in the model; if *γ*_*s*_ = 0, it indicates otherwise. Similarly, *η*_*r*_ = 1 indicates that a group of rare variants in a defined region (typically a gene) is included in the model; *η*_*r*_ = 0 indicates otherwise. λ_*rj*_ = 1 indicates an individual rare variant *j* at group *r* is included in the model; λ_*rj*_ = 0 indicates otherwise. To make the variable selection more flexible, we assume beta priors on *π*_*i*_,

$$\pi_{i}∼\text{Beta}\left(a_{i},b_{i}\right),$$

with hyper-parameters *a*_*i*_ and *b*_*i*_, *i* = 1, 2, 3. This creates Beta-Bernoulli prior on all inclusion indicators. We use the hyper-parameters to balance power and multiplicity correction for the number of variants similar to [29]. Specifically, we use *a*_*i*_ = 1(*i* = 1, 2, 3), and set *b*_1_ as the total number of common variants, *b*_2_ as the total number of groups of rare variants, and *b*_3_ as the total number of rare variants.

Conditional on the selection indicator, the prior for the coefficient of a common variant, *β*_*s*_, is defined as a normal distribution,

$$\begin{array}{c} p\left(\beta_{s}|\gamma_{s}\right)=N\left(0,d_{s}\right) \end{array}$$ (9)

where *d*_*s*_ is defined by the corresponding *γ*_*s*_:

$$d_{s}=\{\begin{array}{cc} & v_{1}\;if\;\gamma_{s}=1,\\ & v_{0}\;if\;\gamma_{s}=0. \end{array}\;s=1,2,\cdots ,S.$$

Here, we set *v*_0_ as a very small positive value that has the effect of restricting the value of *β*_*s*_ to be close to 0 when the SNP is not selected and *v*_1_ (*v*_1_ > 0) large to allow the *β*_*s*_ coefficient to be estimated. Defining the prior on *β*_*s*_ in this way results in marginal normal mixture distributions that are defined by inclusion, typical of common Bayesian variable selection paradigms [29, 42, 43]. For each common variant, *β*_*s*_ is distributed as a normal distribution with the following mean and variance

$$\mu_{s}=0,\;\text{and}\;\text{var}\left(\beta_{s}\right)=\left(1-\gamma_{s}\right)v_{0}+\gamma_{s}v_{1}.$$

For the rare variant coefficient *α* = (*α*_11_, *α*_12_, ⋯, *α*_*rj*_, ⋯)′, we proposed:

$$\begin{array}{c} p\left(\alpha_{rj}|\eta_{r},\lambda_{rj}\right)∼\left(1-\eta_{r}\lambda_{rj}\right)N\left(0,v_{2}\right)+\eta_{r}\lambda_{rj}N\left(0,v_{3}\right), \end{array}$$ (10)

where *r* is the index of region, *r* = 1, 2, ⋯, *R*; and *j* is the index of individual rare variant in the region *r*, *j* = 1, 2, ⋯, *J*_*r*_; *η*_*r*_ is the binary selection indicator of region *r*, λ_*rj*_ is the binary selection indicator of individual rare variant *j* in region *r*; *v*_2_ is the exclusion parameter of individual rare variant when either *η*_*r*_ = 0 or λ_*rj*_ = 0, *v*_3_ is the inclusion parameter of individual rare variant when both *η*_*r*_ = 1 and λ_*rj*_ = 1.

### Posterior inference using the EM algorithm

Let *γ*, *η*, and λ denote the collections of binary selection indicators for individual common variants, regions, and individual rare variants, *γ* = {*γ*_*s*_, *s* = 1, 2, ⋯, *S*}, *η* = {*η*_*r*_, *r* = 1, 2, ⋯, *R*}, and λ = {λ_*rj*_, *r* = 1, 2, ⋯, *R*;*j* = 1, 2, ⋯, *J*_*r*_}. The full posterior distribution is denoted as log*P*(*β*, *α*, *π*_1_, *π*_2_, *π*_3_, *γ*, *η*, λ|*g*, *G*_*m*_, *G*_*f*_, *D*^+^). Given the likelihood of Eq (7), and priors of Eqs (8) to (10) as defined above, the posterior distribution does not have a closed form. Instead of simulating large samples directly from the posterior using Markov Chain Monte Carlo (MCMC) methods, we employ the expectation maximization (EM) algorithm to estimate the posterior modes of interest. In the EM algorithm, we treat the variable selection indicators *γ*, *η*, and λ as missing data and alternate between conditional expectation using the current best estimates for the parameters and maximization of the expectation of the complete log-likelihood (Q function) to estimate the posterior modes of *β* and *α* [43].

For the E-step, we determine the Q-function which is the conditional expectation of log-likelihood of complete data with respect to the missing indicator variables, *γ*, *η*, and λ, given the current estimates of the unknown parameters $\beta^{\left(k\right)},\alpha^{\left(k\right)},\pi_{1}^{\left(k\right)}$, $\pi_{2}^{\left(k\right)},\pi_{3}^{\left(k\right)}$, where *k* is the index of current iteration. For the M-step, we maximize the Q-function with respect to the parameters *β*, *α*, *π*_1_, *π*_2_, *π*_3_ and iterate both steps until convergence. For iteration *k*, the Q-function is defined as:

$$\begin{array}{c} \begin{array}{cc} & Q[\beta ,\alpha ,\pi_{1},\pi_{2},\pi_{3}|\beta^{\left(k\right)},\alpha^{\left(k\right)},\pi_{1}^{\left(k\right)},\pi_{2}^{\left(k\right)},\pi_{3}^{\left(k\right)}]\\ & =E_{\gamma ,\eta ,\lambda |.}[\text{log}\;L(\beta ,\alpha ,\pi_{1},\pi_{2},\pi_{3},\gamma ,\eta ,\lambda |g,G_{m},G_{f},D^{+})] \end{array} \end{array}$$ (11)

where $E_{\gamma ,\eta ,\lambda |.}=E_{\gamma ,\eta ,\lambda |\beta^{\left(k\right)},\alpha^{\left(k\right)},\pi_{1}^{\left(k\right)},\pi_{2}^{\left(k\right)},\pi_{3}^{\left(k\right)},g,G_{m},G_{f},D^{+}}$, where the distributions of *γ*, *η*, λ are given by Eq (8), and *L*(*β*, *α*, *π*_1_, *π*_2_, *π*_3_, *γ*, *η*, λ|**g**, **G**_*m*_, **G**_*f*_, *D*^+^) is the likelihood of complete data which is given by Eq (7).

#### E-step

The objective function Q can be simplified as the sum of conditional functions

$$\begin{array}{c} \begin{array}{cc} Q(\cdot ) & =C+Q_{1}[\beta ,\alpha |\beta^{\left(k\right)},\alpha^{\left(k\right)},\pi_{1}^{\left(k\right)},\pi_{2}^{\left(k\right)},\pi_{3}^{\left(k\right)}]+Q_{2}[\pi_{1}|\beta^{\left(k\right)},\alpha^{\left(k\right)},\pi_{1}^{\left(k\right)}]\\ & +Q_{3}[\pi_{2}|\beta^{\left(k\right)},\alpha^{\left(k\right)},\pi_{2}^{\left(k\right)}]+Q_{4}[\pi_{3}|\beta^{\left(k\right)},\alpha^{\left(k\right)},\pi_{3}^{\left(k\right)}], \end{array} \end{array}$$ (12)

where *C* is constant term and each *Q*_1_, *Q*_2_, *Q*_3_, *Q*_4_ can be maximized independently. For convenience, we index the genotype of cases in each family as *g*_0*n*_, and all the genotypes of pseudo-siblings are indexed by *i* ∈ {1, 2, 3}. Then, the common and rare SNPs of the case child from family *n* are denoted as *g*_*c*0*n*_ and *g*_*r*0*n*_, respectively; and the common and rare SNPs of pseudo-siblings from family *n* are denoted as *g*_*cin*_ and *g*_*rin*_, *i* = 1, 2, 3, respectively. In total of *N* families, according to the likelihood function Eqs (6) and (7), the Q-function with respect to *β* and *α* can be written as

$$\begin{array}{c} \begin{array}{cc} & Q_{1}[\beta ,\alpha |\beta^{\left(k\right)},\alpha^{\left(k\right)},\pi_{1}^{\left(k\right)},\pi_{2}^{\left(k\right)},\pi_{3}^{\left(k\right)}]\\ & =\sum_{n=1}^{N}\;\text{log}[\frac{exp(g_{c0n}\beta +g_{r0n}\alpha )}{exp\left(g_{c0n}\beta +g_{r0n}\alpha \right)+\sum_{i=1}^{3}exp\left(g_{cin}\beta +g_{rin}\alpha \right)}]\\ & -\frac{1}{2}\sum_{s=1}^{S}\beta_{s}^{2}E_{\gamma_{s}|.}[\frac{1}{v_{0}\left(1-\gamma_{s}\right)+v_{1}\gamma_{s}}]\\ & -\frac{1}{2}\sum_{r=1}^{R}\sum_{j=1}^{J_{r}}\alpha_{rj}^{2}E_{\eta_{r},\lambda_{rj}|.}[\frac{1}{v_{2}\left(1-\eta_{r}\lambda_{rj}\right)+v_{3}\eta_{r}\lambda_{rj}}]\\ & =-\sum_{n=1}^{N}\;\text{log}\left(1+\sum_{i=1}^{3}e^{-x_{cin}\beta -x_{rin}\alpha }\right)-\frac{1}{2}\beta^{\prime }P_{c}^{\left(k\right)}\beta -\frac{1}{2}\alpha^{\prime }P_{r}^{\left(k\right)}\alpha \end{array} \end{array}$$ (13)

where *x*_*in*_ = *g*_0*n*_ − *g*_*in*_ which is a 1 × *p* vector; $P_{c}^{\left(k\right)}$ is a *S*×*S* diagonal matrix with elements $\left(1-p_{s}^{\left(k\right)}\right)\frac{1}{v_{0}}+p_{s}^{\left(k\right)}\frac{1}{v_{1}},s\in \left\{1,2,\ldots ,S\right\}$, and *S* is the total number of the common variants, $p_{s}^{\left(k\right)}$ is the conditional expectation of inclusion parameter. Based on Bayes’ rule, $p_{s}^{\left(k\right)}$ can be calculated as follows.

$$\begin{array}{c} p_{s}^{\left(k\right)}=E_{\gamma_{s}|.}[\gamma_{s}]=P\left(\gamma_{s}=1|\beta^{\left(k\right)},\pi_{1}^{\left(k\right)}\right)=\frac{a_{s}}{a_{s}+b_{s}}, \end{array}$$ (14)

where $a_{s}=P\left(\beta^{\left(k\right)}|\gamma_{s}=1\right)P\left(\gamma_{s}=1|\pi_{1}^{\left(k\right)}\right)$, $b_{s}=P\left(\beta_{s}^{\left(k\right)}|\gamma_{s}=0\right)P\left(\gamma_{s}=0|\pi_{1}^{\left(k\right)}\right)$ and $P\left(\gamma_{s}=1|\pi_{1}^{\left(k\right)}\right)=\pi_{1}^{\left(k\right)}$. $P_{r}^{\left(k\right)}$ is a *L* × *L* diagonal matrix with elements $\left(1-q_{rj}^{\left(k\right)}\right)\frac{1}{v_{2}}+q_{rj}^{\left(k\right)}\frac{1}{v_{3}}$, *r* = 1, 2, ⋯, *R*, *j* = 1, 2, ⋯, *J*_*r*_, *L* is the total number of rare variants, *R* is the number of groups (regions) of variants, *J*_*r*_ is the number of rare variants in the group (region) *r*, and

$$\begin{array}{c} \begin{array}{c} q_{rj}^{\left(k\right)}=E_{\lambda_{rj=1}|\eta_{r}=1,\cdots }\left[\lambda_{rj}\right]=P\left(\lambda_{rj}=1|\eta_{r}=1,\alpha_{rj}^{\left(k\right)},\pi_{2}^{\left(k\right)},\pi_{3}^{\left(k\right)}\right)=\frac{c_{rj}}{c_{rj}+d_{rj}}, \end{array} \end{array}$$ (15)

where $c_{rj}=\pi_{3}^{\left(k\right)}P\left(\alpha_{rj}^{\left(k\right)}|\lambda_{rj}=1,\eta_{r}=1\right)$, and $d_{rj}=\left(1-\pi_{3}^{\left(k\right)}\right)P\left(\alpha_{rj}^{\left(k\right)}|\lambda_{rj}=0,\eta_{r}=1\right)$. The second and third terms of the Q function can be calculated as

$$\begin{array}{c} Q_{2}[\pi_{1}|\beta^{\left(k\right)},\pi_{1}^{\left(k\right)}]=\sum_{s=1}^{S}\;E_{\gamma_{s}|.}[\gamma_{s}]\text{log}[\frac{\pi_{1}}{1-\pi_{1}}]+\left(2S-1\right)\text{log}\left(1-\pi_{1}\right). \end{array}$$ (16)

$$\begin{array}{c} Q_{3}[\pi_{2}|\alpha^{\left(k\right)},\pi_{2}^{\left(k\right)}]=\sum_{r=1}^{R}\;E_{\eta_{r}|.}\left[\eta_{r}\right]\text{log}[\frac{\pi_{2}}{1-\pi_{2}}]+\left(2R-1\right)\text{log}\left(1-\pi_{2}\right), \end{array}$$ (17)

where

$$\begin{array}{c} E_{\eta_{r}|\cdot }\left[\eta_{r}\right]=P\left(\eta_{r}=1|\alpha_{r1}^{\left(k\right)},\cdots ,\alpha_{rJ_{r}}^{\left(k\right)},\pi_{2}^{\left(k\right)},\pi_{3}^{\left(k\right)}\right)=\frac{e_{r}}{e_{r}+f_{r}}≐g_{r}^{\left(k\right)}, \end{array}$$ (18)

in which *e*_*r*_ and *f*_*r*_ are defined as

$$e_{r}=\pi_{2}^{\left(k\right)}\prod_{j=1}^{J_{r}}[\pi_{3}^{\left(k\right)}P\left(\alpha_{rj}^{\left(k\right)}|\lambda_{rj}=1,\eta_{r}=1\right)+\left(1-\pi_{3}^{\left(k\right)}\right)P\left(\alpha_{rj}^{\left(k\right)}|\lambda_{rj}=0,\eta_{r}=1\right)],$$

and

$$f_{r}=\left(1-\pi_{2}^{\left(k\right)}\right)\prod_{j=1}^{J_{r}}\;P\left(\alpha_{rj}^{\left(k\right)}|\lambda_{rj}=0,\eta_{r}=0\right).$$

The last term of the Q function is written as

$$\begin{array}{c} \begin{array}{cc} & Q_{4}[\pi_{3}|\alpha^{\left(k\right)},\pi_{2}^{\left(k\right)},\pi_{3}^{\left(k\right)}]=E_{\eta ,\lambda |\cdot }\text{log}[P\left(\pi_{3}\right)\prod_{r=1}^{R}\;\prod_{j=1}^{J_{r}}P\left(\lambda_{rj}|\eta_{r},\pi_{3}\right)]\\ & =\sum_{r=1}^{R}g_{r}^{\left(k\right)}\sum_{j=1}^{J_{r}}q_{rj}^{\left(k\right)}\text{log}\left(\frac{\pi_{3}}{1-\pi_{3}}\right)+\left(L+\sum_{r=1}^{R}g_{r}^{\left(k\right)}J_{r}-1\right)\text{log}\left(1-\pi_{3}\right) \end{array} \end{array}$$ (19)

#### M-step

For the M-step, we maximize *Q*_1_, *Q*_2_, *Q*_3_ and *Q*_4_ separately. There is no closed-form solution for *Q*_1_ function. However, maximizing the *Q*_1_ with respect to *β* and *α* is equivalent to a minimization problem with respect to parameter *ω* (*p* × 1), where *p* = *S* + *L*, *S* is the total number of common variants, and *L* is the total number of rare variants. Based on Eq (13),

$$\begin{array}{c} \begin{array}{cc} & \underset{\beta ,\alpha }{\text{max}}\;Q_{1}[\beta ,\alpha |\beta^{\left(k\right)},\alpha^{\left(k\right)},\pi_{1}^{\left(k\right)},\pi_{2}^{\left(k\right)},\pi_{3}^{\left(k\right)}]\\ & =\underset{\beta ,\alpha }{\text{max}}(-\sum_{n=1}^{N}\;\text{log}\left(1+\sum_{i=1}^{3}\;e^{-x_{cin}\beta -x_{rin}\alpha }\right)-\frac{1}{2}\beta^{\prime }P_{c}^{\left(k\right)}\beta -\frac{1}{2}\alpha^{\prime }P_{r}^{\left(k\right)}\alpha )\\ & =\underset{\beta ,\alpha }{\text{min}}(\sum_{n=1}^{N}\;\text{log}\left(1+\sum_{i=1}^{3}\;e^{-x_{cin}\beta -x_{rin}\alpha }\right)+\frac{1}{2}\beta^{\prime }P_{c}^{\left(k\right)}\beta +\frac{1}{2}\alpha^{\prime }P_{r}^{\left(k\right)}\alpha )\\ & =\underset{\omega }{\text{min}}(\sum_{n=1}^{N}\;\text{log}\left(1+\sum_{i=1}^{3}\;e^{-x_{in}\omega }\right)+\frac{1}{2}\omega^{\prime }P^{\left(k\right)}\omega ), \end{array} \end{array}$$ (20)

where *x*_*in*_ = (*x*_*cin*_, *x*_*rin*_), $\omega ={\left(\beta_{1},\beta_{2},\ldots ,\beta_{S},\alpha_{11},\alpha_{12},\ldots ,\alpha_{rj},\ldots ,\alpha_{RJ_{R}}\right)}^{\prime },$ *P*^(*k*)^ is a *p* × *p* (*p* = *S* + *L*) diagonal matrix with diagonal elements $\left(1-p_{s}^{\left(k\right)}\right)\frac{1}{v_{0}}+p_{s}^{\left(k\right)}\frac{1}{v_{1}},s\in \left\{1,2,\ldots ,S\right\})$ for the first *S* elements, and $\left(1-q_{rj}^{\left(k\right)}\right)\frac{1}{v_{2}}+q_{rj}^{\left(k\right)}\frac{1}{v_{3}}$ for the rest of L elements.

Since the likelihood function of conditional logistic regression and $\frac{1}{2}\omega^{\prime }P^{\left(k\right)}\omega$ are vector convex functions [43], we used stochastic dual coordinate ascent (SDCA) [45, 46], an efficient technique for solving regularized loss minimization problems in machine learning, to solve the minimization problem above. Particularly, the accelerated min-batch SDCA was implemented and the details of the algorithm can be found in the Supplemental Materials [46]. Accordingly, we compute the *β* and *α* estimates for the next iteration based on the *Q*_1_ function. The remaining components *Q*_2_, *Q*_3_, and *Q*_4_ have closed forms. The details of solving the closed form solutions to the maximization of *Q*_2_, *Q*_3_, and *Q*_4_ are shown in the Supplemental Materials. The closed form solution for *Q*_2_ is:

$$\begin{array}{c} \pi_{1}^{\left(k+1\right)}=\frac{\sum_{s=1}^{S}p_{s}^{\left(k\right)}}{2S-1} \end{array}$$ (21)

The closed form solution for *Q*_3_ is:

$$\begin{array}{c} \pi_{2}^{\left(k+1\right)}=\frac{\sum_{r=1}^{R}g_{r}^{\left(k\right)}}{2R-1} \end{array}$$ (22)

The closed form solution for *Q*_4_ is:

$$\begin{array}{c} \pi_{3}^{\left(k+1\right)}=\frac{\sum_{r=1}^{R}\;g_{r}^{\left(k\right)}\sum_{j=1}^{J_{r}}q_{rj}^{\left(k\right)}}{L+\sum_{r=1}^{R}\;g_{r}^{\left(k\right)}J_{r}-1} \end{array}$$ (23)

Convergence of the algorithm is determined if the difference between two successive observed-data likelihood is less than *ϵ*, i.e.

$$\begin{array}{c} \begin{array}{cc} & l\left(\theta^{\left(k+1\right)}|y_{obs}\right)-\left(\theta^{\left(k\right)}|y_{obs}\right)\\ & =[Q\left(\theta^{\left(k+1\right)},\theta^{\left(k\right)}\right)-Q\left(\theta^{\left(k\right)},\theta^{\left(k\right)}\right)]-[R\left(\theta^{\left(k+1\right)},\theta^{\left(k\right)}\right)-R\left(\theta^{\left(k\right)},\theta^{\left(k\right)}\right)]<\epsilon , \end{array} \end{array}$$ (24)

where *ϵ* is a pre-specified threshold, *θ* = (*β*, *α*, *π*_1_, *π*_2_, *π*_3_), and

$$\begin{array}{c} \begin{array}{cc} & R[\beta ,\alpha ,\pi_{1},\pi_{2},\pi_{3}|\beta^{\left(k\right)},\alpha^{\left(k\right)},\pi_{1}^{\left(k\right)},\pi_{2}^{\left(k\right)},\pi_{3}^{\left(k\right)}]\\ & =E_{\gamma ,\eta ,\lambda |.}[\text{log}\;P(\gamma ,\eta ,\lambda |g,G_{m},G_{f},D^{+},\beta ,\alpha ,\pi_{1},\pi_{2},\pi_{3})]\\ & =\sum_{s=1}^{S}\;E_{\gamma_{s}|.}[\gamma_{s}]\text{log}[\frac{\pi_{1}}{1-\pi_{1}}]+S\;\text{log}\left(1-\pi_{1}\right)\\ & +\sum_{r=1}^{R}\;E_{\eta_{r}|.}\eta_{r}\;\text{log}[\frac{\pi_{2}}{1-\pi_{2}}]+R\;\text{log}\left(1-\pi_{2}\right)\\ & +\sum_{r=1}^{R}\;g_{r}^{\left(k\right)}\sum_{j=1}^{J_{r}}\;q_{rj}^{\left(k\right)}\;\text{log}\left(\frac{\pi_{3}}{1-\pi_{3}}\right)+\left(\sum_{r=1}^{R}\;g_{r}^{\left(k\right)}J_{r}\right)\text{log}\left(1-\pi_{3}\right). \end{array} \end{array}$$ (25)

### Deterministic annealing

Though the conventional EM algorithm has attractive features, it can become trapped in local maximums in multimodal posterior distributions. To enhance the chance of discovering a global mode, the Deterministic Annealing variant of the EM algorithm (DAEM) is considered [47]. During each DAEM iteration, the conditional probability of inclusion indicators is parameterized by temperature 1/*t* (0 < *t* < 1). Therefore, $p_{s}^{\left(k\right)}$, $q_{rj}^{\left(k\right)}$ and $g_{r}^{\left(k\right)}$ from Eqs (14), (15) and (18) were substituted by:

$$\begin{array}{c} p_{s}^{\left(k\right)}=\frac{a_{s}^{t}}{a_{s}^{t}+b_{s}^{t}} \end{array}$$ (26)

$$\begin{array}{c} q_{rj}^{\left(k\right)}=\frac{c_{rj}^{t}}{c_{rj}^{t}+d_{rj}^{t}} \end{array}$$ (27)

$$\begin{array}{c} g_{r}^{\left(k\right)}=\frac{e_{r}^{t}}{e_{r}^{t}+f_{r}^{t}} \end{array}$$ (28)

where *a*_*s*_, *b*_*s*_, *c*_*rj*_, *d*_*rj*_, *e*_*r*_ and *f*_*r*_ are the same as in Eqs (14), (15) and (18). In this study, for both simulation and real-data analysis, the initial value of coefficients of common and rare variants is 0.5; the initial value of *t* is 0.1 with an incremental value of 0.1.

### Selection parameter tuning

Here, we present a recommendation for tuning the selection parameters in TRIO_RVEMVS. Specifically, selection is controlled through the exclusion parameters, *v*_0_ and *v*_2_, and inclusion parameters, *v*_1_ and *v*_3_, as similar to [43]. To determine suitable values for these parameters, we first considered an odds ratio between [0.95,1.05] to be clinically irrelevant. Then, given a 95% prior probability of variable inclusion of an odds ratio that covers [0.29,3.45] and [0.27,4.3] for common and rare variants respectively. Thus, we set *v*_1_ = 0.4 and *v*_3_ = 0.5. Next, we evaluated the local stability of regularization plots with respect to exclusion parameters. The tuning process proceeded as follows:

- We initially set the exclusion parameters for common and rare variants to be equal, i.e. *v*_0_ = *v*_2_, and chose the common exclusion parameter in the local stable regularization plot window.
- Subsequently, with the common exclusion parameter fixed, we evaluated the local stability of the regularization plot with respect to the rare variant exclusion parameter *v*_2_, and chose the *v*_2_ within a stable window defined by at least 3 points in the grid where no shrinkage occurs.

This procedure was applied in both simulated and real data analyses.

## Simulation

We simulated two scenarios consisting of 500 datasets each to evaluate the performance of TRIO_RVEMVS in identifying regions/genes of interest relative to existing methods (PedGene [37] and RV-TDT [38]) as well as its ability to identify individual variants. One scenario generates 1500 case-parent trios per data set, while the other involves generating 350 case-parent trios per dataset. These scenarios allowed us to assess the performance of TRIO_RVEMVS under large-sample and small-sample conditions, respectively.

To measure the overall performance of region detection, we calculated the weighted average correct association percentage with

$$\begin{array}{c} \frac{1}{2}[\frac{\sum P(\text{selected}|\text{associated})}{\text{total}\;\text{number}\;\text{of}\;\text{associated}\;\text{regions}}+\frac{\sum P(\text{unselected}|\text{unassociated})}{\text{total}\;\text{number}\;\text{of}\;\text{unassociated}\;\text{regions}}] \end{array}$$ (29)

Considering that most of the rare variants were not polymorphic across all data sets, we defined the Average True and False Positive Rate (ATPR and AFPR) for individual variants as follows:

$$\begin{array}{c} \begin{array}{cc} & \text{ATPR}=\frac{1}{\#\;\text{of}\;\text{data}\;\text{sets}}\sum_{\text{data}\;\text{set}\;d}\frac{N_{d}\left(\text{selected}|\text{associated}\right)}{N_{d}\left(\#\;\text{of}\;\text{polymorphic}\;\text{associated}\;\text{variants}\right)}\\ & \text{AFPR}=\frac{1}{\#\;\text{of}\;\text{data}\;\text{sets}}\sum_{\text{data}\;\text{set}\;d}\frac{N_{d}\left(\text{selected}|\text{unassociated}\right)}{N_{d}\left(\#\;\text{of}\;\text{polymorphic}\;\text{unassociated}\;\text{variants}\right)} \end{array} \end{array}$$ (30)

where *N*_*d*_(selected|⋅) denotes the number of detected variants given the variants are associated or unassociated in data set *d*.

### Data simulation

We simulated the population of haplotypes using Cosi2 [48], which is a forward-time genetic simulator. We used the 1000 Genomes Project [49] haplotypes as the reference population for Cosi2 and simulated a 30kb region of chromosome 1, consisting of 45965 SNPs. We simulated populations of 80,000 African haplotypes and 80,000 European haplotypes. Then we constructed 500 samples, each consisting of 60,000 haplotypes (15,000 African and 45,000 European, reflecting 25% and 75%, respectively) from the simulated population (with replacement). For each of the 500 samples of haplotypes, we randomly selected and paired haplotypes within the race to construct individuals, and then randomly paired individuals within the race to construct parents. Then from each set of parents, we randomly selected one haplotype from each parent to be transmitted to the child to form 15,000 trios. The full simulation algorithm is described in the Supplemental Materials.

We defined a gene region as 2700 base pairs, resulting in 12 simulated gene regions. Within gene regions, we used population allele frequencies to determine rare and common variants. Since we simulated admixed samples, we computed a weighted minor allele frequency (MAF) for each SNP. This was based on the frequency estimates from our reference genome (the 1000 Genomes Project) for each population (African and European), weighted by the proportion of each population admixed for our simulation (25% African and 75% European). Rare variants were defined as variants with a weighted MAF < 0.05. For simplicity, we simulated our causal SNPs on genes 1–6. We modeled disease based on two causal common SNPs (risk-increasing allele on gene 3 and risk-decreasing allele on gene 6) and 5% of any variant with weighted MAF less than 3% were randomly chosen as causal rare variants. In total, 1212 rare variants were simulated as causal to the simulated disease, with 606 simulated as risk increasing and 606 as risk decreasing. The distribution of associated rare variants across the 6 genes is reported in Table 1.

**Table 1:** Number of associated rare variants with different range of weighted MAF by region in the haplotype pool. Across all simulated data sets, most of the variants with weighted MAF less than 0.0001 were those non-polymorphic, singleton, doubletons, or triptons.

Region	(0, 0.0001)	[0.0001, 0.001)	[0.001, 0.01)	[0.01, 0.05)
1	204	4	3	1
2	172	2	0	0
3	204	3	2	0
4	220	5	0	0
5	197	2	1	0
6	185	6	1	0
Total	1182	22	7	1

We simulated the disease status of each child according to the logistic model:

$$\begin{array}{c} \text{logit}\left(P\left(y=1\right)\right)=\alpha_{0}+\beta_{1}G_{1}^{c}+\beta_{2}G_{2}^{c}+\cdots +\beta_{p}G_{p}^{c}, \end{array}$$ (31)

where $G_{1}^{c},G_{2}^{c},\ldots ,G_{p}^{c}$ were the children’s genotypes for the *p* causal variants (consisting of the 2 common variants, and the rest are rare variants), and we set *α*_0_ = −2.2 to control the disease incidence to be low. For the simulation, we set the magnitude of the coefficients for our causal common variants to be 0.9, and the magnitude of the coefficients for causal rare variants was computed as in [28]: *c*|*log*_10_*MAF*_*i*_|, where *c* = 0.4 for causal risk rare variants, and *c* = −0.4 for causal protective rare variants, and *MAF*_*i*_ is the weighted MAF of locus *i*. Assigning the coefficients in this way results in rarer variants having a stronger effect on disease.

The distribution of associated rare variants across 6 regions is shown in Table 1. After simulating diseased probands in each set of 15,000 trios, we generated 500 replicates of 1,500 case-parent trios, and another 500 replicates of 350 case-parent trios to assess performance for large and small sample sizes, respectively.

Due to sampling, some loci that were polymorphic in the population with rare variants became non-polymorphic in the 500 samples, and the number of polymorphic loci varied across the 500 data sets. Some of the polymorphic loci with rare variants that were in the disease-generating model were not polymorphic in one or more samples. The distribution of polymorphic variants across the 500 data sets for each sample size is depicted in Table 2.

**Table 2:** Summary statistics of data sets with 1,500 and 350 case-trios.

Summary statistics	1500 case-trios	350 case-trios
Average # polymorphic variants	4008	1442
Average # polymorphic common variants	48	47
Average # polymorphic causal common variants	2	2
Average # polymorphic rare variants	3960	1395
Average # polymorphic causal rare variants	132	45
# polymorphic variants across 500 data sets	431	133
# polymorphic causal variants across 500 data sets	12	4

### Analysis of simulated data

We used the same analysis strategy across all simulated datasets. First, we classified variants as either common or rare in each simulated data set. We estimated the MAF of each variant in each dataset for each sample size. For each sample size, we defined rare variants based on the median MAF across the 500 data sets, with the threshold of rare variants being median MAF < 5%. To assess performance in identifying regions of interest, we applied all three methods (TRIO_RVEMVS, PedGene, and RV-TDT) to the 500 data sets, summarizing the performance of each method using the weighted average correct association metric. Since only TRIO_RVEMVS identifies specific rare variants, we also report a similar weighted correct association metric for individual rare variants. For TRIO_RVEMVS, the detailed exclusion parameter tuning using regularization plot can be found in the Supplemental Materials.

### Simulation results

In this section, we first compare selection at the region level with PedGene [37] and RV-TDT [38] with the weighted average correct association percentage. Specifically, we compared TRIO_RVEMVS with PedGene kernel and burden methods [37]. For RV-TDT, we evaluated different variants, such as BRV-Haplo, VT-BRV-Haplo, WSS-Haplo, CMC-Analytical, CMC-Haplo, and VT-CMC-Haplo [38]. TRIO_RVEMVS outperformed both PedGene and RV-TDT when jointly considering common and rare variants. Our simulation analyses also confirmed that PedGene showed improved performance in detecting rare variants compared to RV-TDT [50]. We conclude our simulation analysis by discussing the capacity of TRIO_RVEMVS to detect individual rare variants, which is not accomplished by either PedGene or RV-TDT.

**Fig 1:**
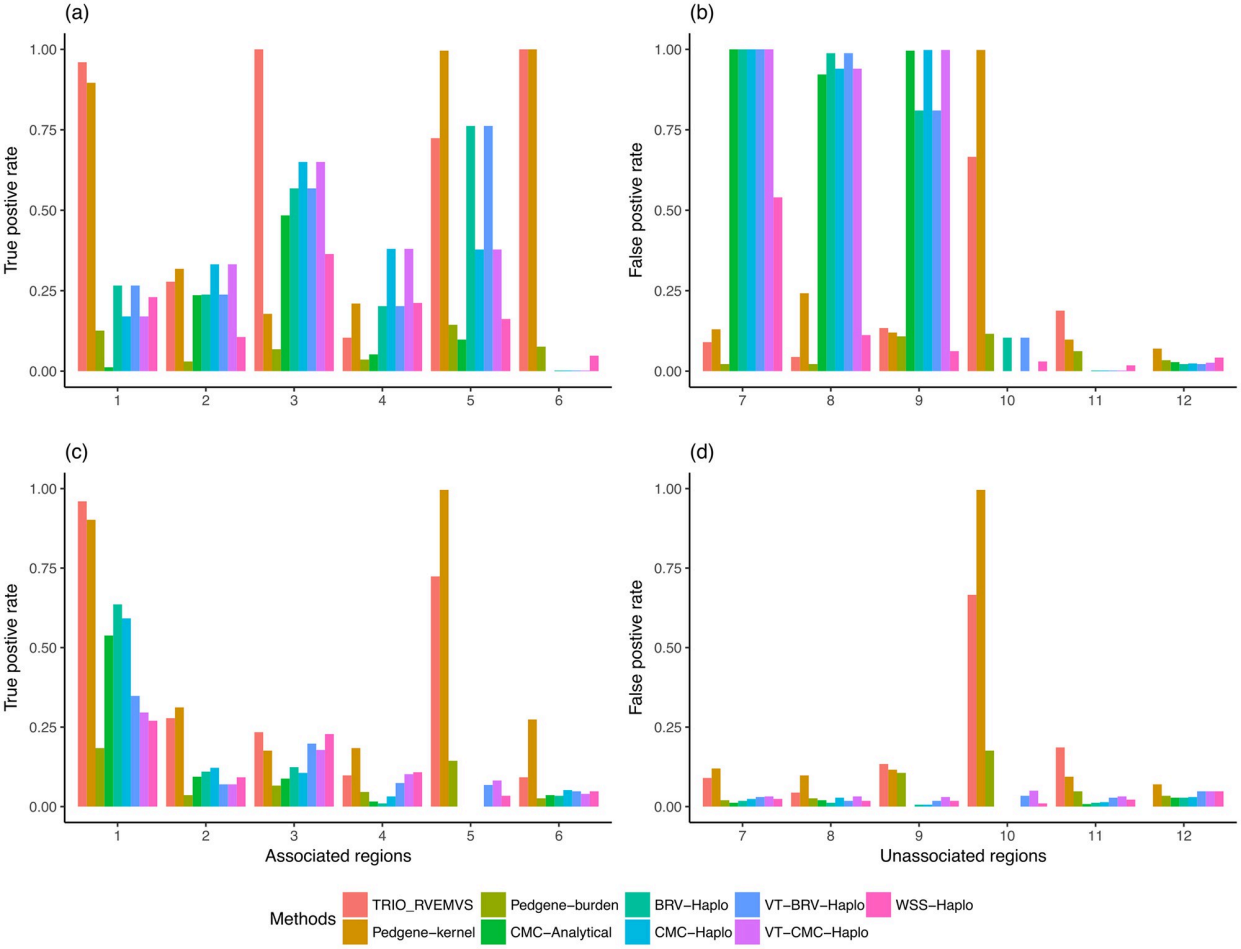
For the data sets with 1,500 case-trios, panels (a) and (b) showed the true and false positive rates of analyzing regions using both common and rare variants; panels (c) and (d) showed the true and false positive rates of regions with rare variants only.

We compared the performance of all methods in two ways. First, we compared each method’s ability to select risk regions based on rare variants only. Second, we compared each method’s ability to identify risk regions when jointly analyzing rare and common variants. For all analyses, we defined rare variants as SNPs whose MAF < 5%. Panels a) and b) of Fig 1 show the true and false positive rate of region selection when jointly analyzing common and rare variants using datasets with 1500 case-trios. In panel a), it shows that TRIO_RVEMVS outperformed both PedGene and RV_TDT with higher true positive rates across the 6 simulated causal regions except for simulated region 5, where PedGene shows a better true positive rate of detection. Considering the false positive rates across regions 7–12, PedGene and Trio_RVEMVS were competitive, TRIO_RVEMVS outperformed PedGene’s false positive rate across regions 7, 8,10, and 12, while PedGene had slightly lower false positive rates for regions 9 and 11. Panels c) and d) of Fig 1 show the true and false positive rates when focusing solely on rare variants detection for data sets with 1500 case-trios. TRIO_RVEMVS outperformed PedGene in regions 1 and 3 in terms of true positive rate but performed just behind PedGene in regions 2, 4, 5, and 6.

Table 3 summarizes the weighted average correct association percentage (WACAP), Eq (29), for TRIO_RVEMVS, RV-TDT, and PedGene. TRIO_RVEMVS shows the highest WACAP when selecting both common and rare variants. When focusing on using rare variants only, TRIO_RVEMVS was competitive with PedGene-Kernel, but did not always have the highest WACAP.

**Table 3:** The comparison of weighted average correct association percentage between TRIO_RVEMVS, PedGene and RV-TDT with and without common variants.

Methods	1500 case-trios		350 case-trios	
	common and rare	rare only	common and rare	rare only
TRIO_RVEMVS	74.53	60.55	66.37	52.07
PedGene-kernel	66.17	61.25	55.73	52.65
PedGene-burden	50.97	50.77	49.90	49.70
CMC-Analytical	32.80	55.86	36.32	52.35
BRV-Haplo	42.60	56.98	40.58	53.08
CMC-Haplo	41.23	56.68	39.00	52.95
VT-BRV-Haplo	42.60	55.25	52.73	52.65
VT-CMC-Haplo	41.21	54.53	57.33	52.10
WSS-Haplo	52.65	55.33	50.18	52.75

**Fig 2:**
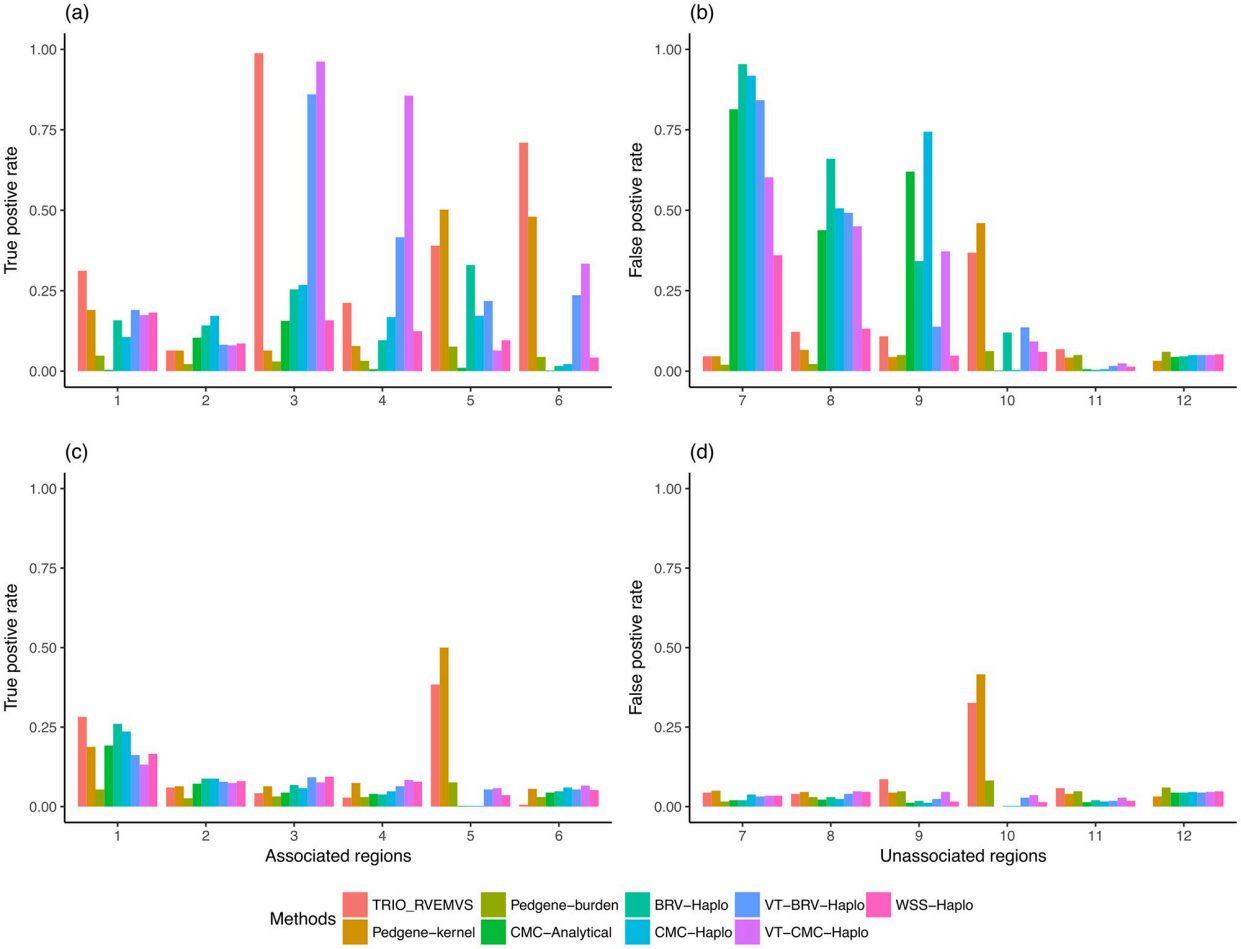
For the data sets with 350 case-trios, panels (a) and (b) showed the true and false positive rates of regions with common variants; panels (c) and (d) showed the true and false positive rates of regions with rare variants only.

In the 350 case-trio data sets, TRIO_RVEMVS outperformed both PedGene and RV_TDT with respect to the weighted average correct association percentage, shown in Table 3. TRIO_RVEMVS achieved the highest average correct association percentage among all methods at 66.37%. VT-CMC-Haplo had the second-highest WACAP at 57.33%. In each region, we observed a consistent pattern of true and false positive rates when analyzing both common and rare variants, Fig 2. Specifically, TRIO_RVEMVS exhibited superior performance in terms of the true positive rate in regions 1, 3, and 6, shown in panel a) in Fig 2. With respect to the false positive rate, TRIO_RVEMVS performed similarly to PedGene; and both have smaller false positive rates compared to RV_TDT. See panel b) in Fig 2. When analyzing only rare variants, all methods demonstrated similar average correct association percentages, as indicated in Table 3. Because of the smaller sample size, all methods showed low power to detect the causal rare variants in general, shown in panels c) and d) in Fig 2. Therefore, compared to the 1500 case-trios, we did not observe notably higher true positive rates or false positive rates at the region level when analyzing only rare variants in the 350 case-trios data analysis.

**Fig 3:**
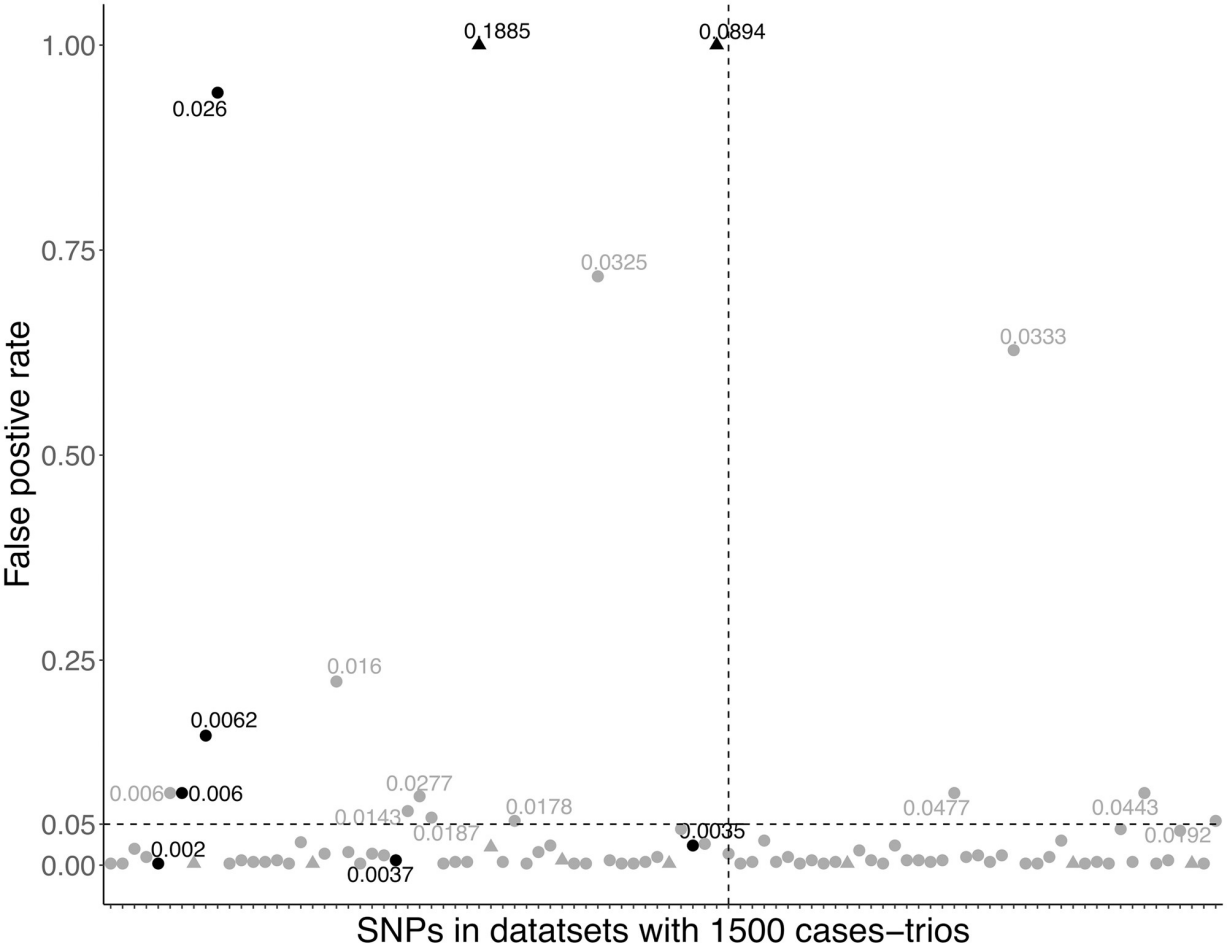
The true and false positive rate of SNPs with corresponding median MAF in 500 data sets with 1500 case-trios respectively. The horizontal dash line represents a threshold of rate 0.05; the vertical line separates the causal and non-causal variants. Variants with black color are true positives; grey illustrates the false positive variants; dot denotes rare variants; triangle denotes common variants.

**Fig 4:**
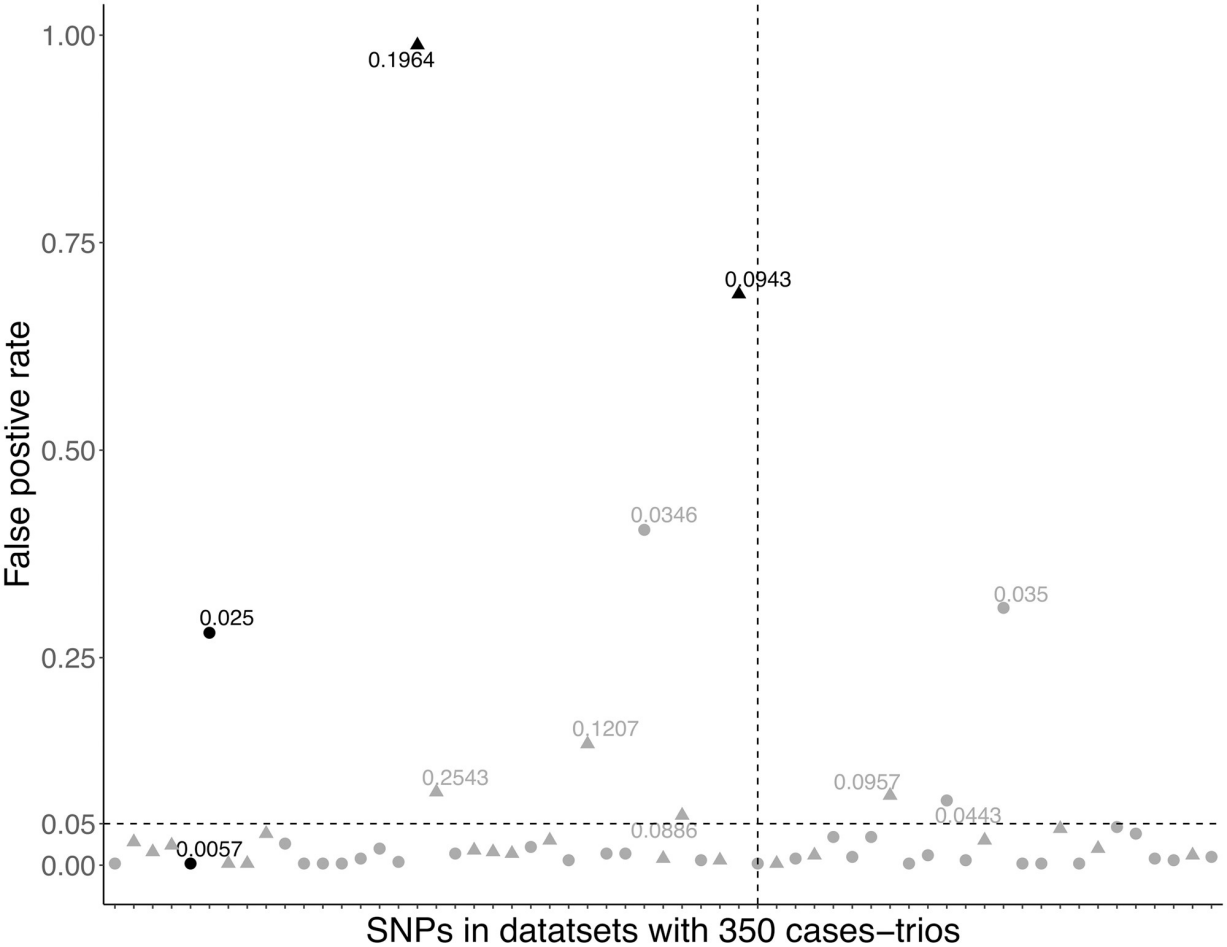
The true and false positive rate of SNPs with corresponding median MAF in 500 data sets with 350 case-trios respectively. The horizontal dash line represents a threshold of rate 0.05; the vertical line separates the causal and non-causal variants. Variants with black color are true positives; grey illustrates the false positive variants; dot denotes rare variants; triangle denotes common variants.

At the individual variants level, TRIO_RVEMVS detected 94 variants including 8 causal in 500 datasets with 1500 case-trios, and 57 variants with 4 causal in 500 datasets with 350 case-trios. The true positive rate (TPR) and false positive rate (FPR) of detected variants are shown in Figs 3 and 4. We primarily focus on reporting the individual-level selection results for variants that were polymorphic across all datasets, due to the low MAF of rare variants, Table 4. When considering the variants that were not all polymorphic across datasets, individual-level selection results were summarized in the Supplemental Materials. For datasets with 1500 case-trios, the ATPRs were 26.83% and 12.20% with and without common variants. The two causal common variants were constantly detected with ATPR of 100%. The AFPRs were 0.67% and 0.74% with and without common variants. For datasets with 350 case-trios, when the common and rare variants were jointly analyzed the ATPR was 48.45%, and the AFPR was 1.38%; when only rare variants were analyzed the ATPR was 13.10% and AFPR was 1.30%. ATPR and AFPR for variants in different ranges of MAF were summarized in Table 4.

**Table 4:** The average true and false positive rate of individual variants detection in different median MAF ranges for variants that were polymorphic across 500 datasets with different sample sizes.

	Sample size	MAF<0.01	0.01≤ MAF<0.05	MAF≥0.05	Total
Number of associated	1500	9	1	2	12
	350	1	1	2	4
ATPR (%)	1500	3.08	94.2	100	26.83
	350	0	26.2	83.8	48.45
Number of unassociated	1500	332	41	46	419
	350	43	40	46	129
AFPR (%)	1500	0.09	6.08	0.09	0.67
	350	0	2.70	1.51	1.38

**Fig 5:**
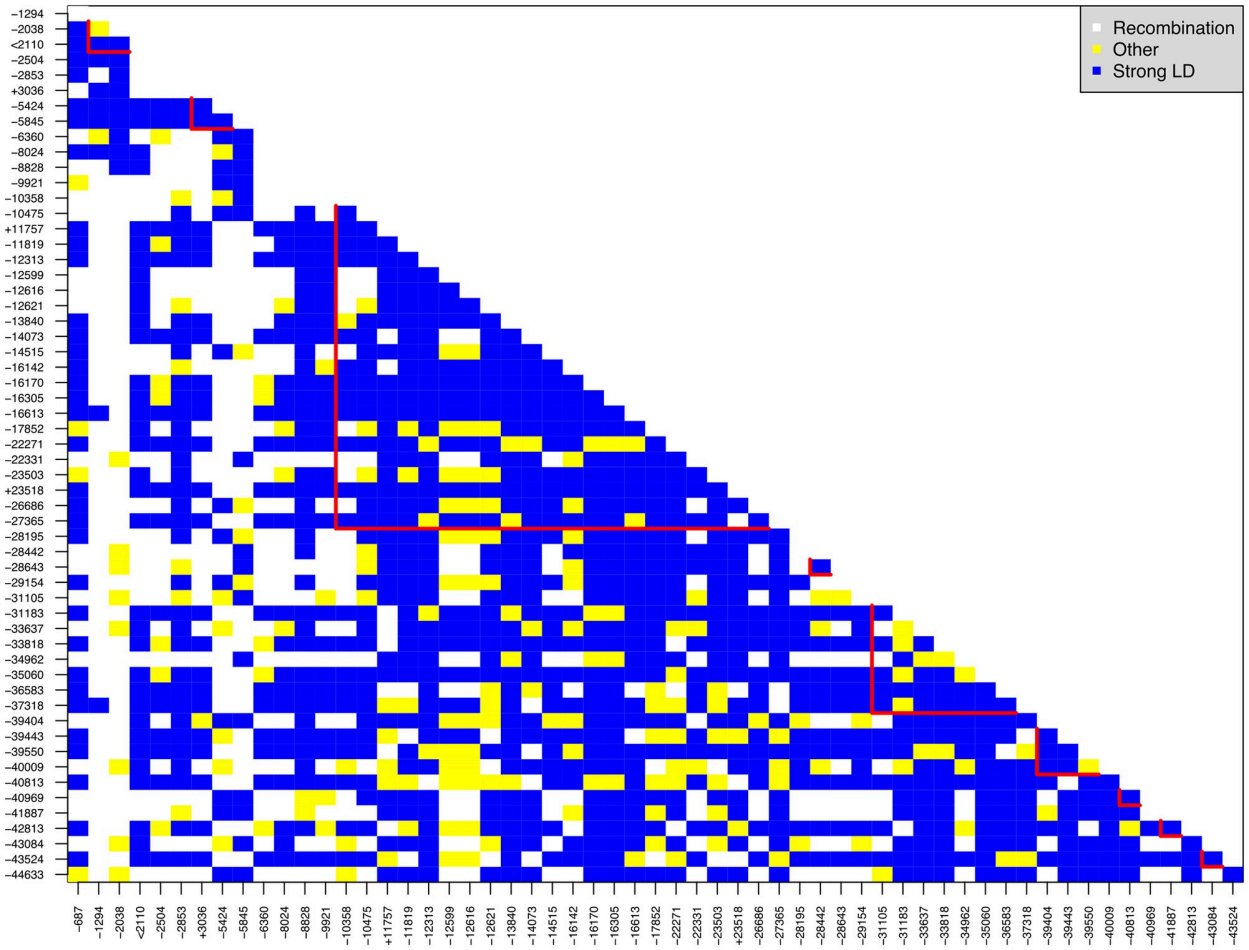
Linkage-disequilibrium (LD) for variants that were polymorphic across 500 data sets with 350 case-trios. The symbol ‘+’ before variant names denote detected associated rare variants; ‘-’ denotes detected non-associated variants; ‘<’ denotes never detected associated variants. V16613 from region 5, and V37318 from region 10 display a high false positive rate, potentially due to LD. They both have strong LD with all the causal rare variants that are polymorphic across 500 data sets.

We observe that the true positive rate is lower and the false positive rate is higher for variants that have lower MAF. We illustrate this using the dataset of 1500 case-trios. For variants with a median MAF less than 0.01, the ATPR and AFPR were 3.08% and 0.09%, respectively. The highest FPR in this group was 8.8%, and the variant with such a high FPR had an equal median MAF to one of the associated variants (0.006) in region 1. In addition, the associated variant in region 1 and this falsely detected unassociated variant was only separated by 53 basepairs, and the linkage disequilibrium between them was 1 (both Dprime and rSquare), Fig 5. The average FPR in the group of variants that had MAF in the range of [0.01, 0.05) was 6.08%. The TPR of the only associated variant (median MAF: 0.026) was 94.2%. Two variants in the same group have relatively high FPR: one rare variant from region 5 had an FPR of 71.8%; another rare variant from region 10 had an FPR of 62.8%. (Those three variants may have contributed the high true and false positive rates, respectively, at the region level for both methods TRIO_RVEMVS and PedGene, Fig 1).

## Real data application

**Fig 6:**
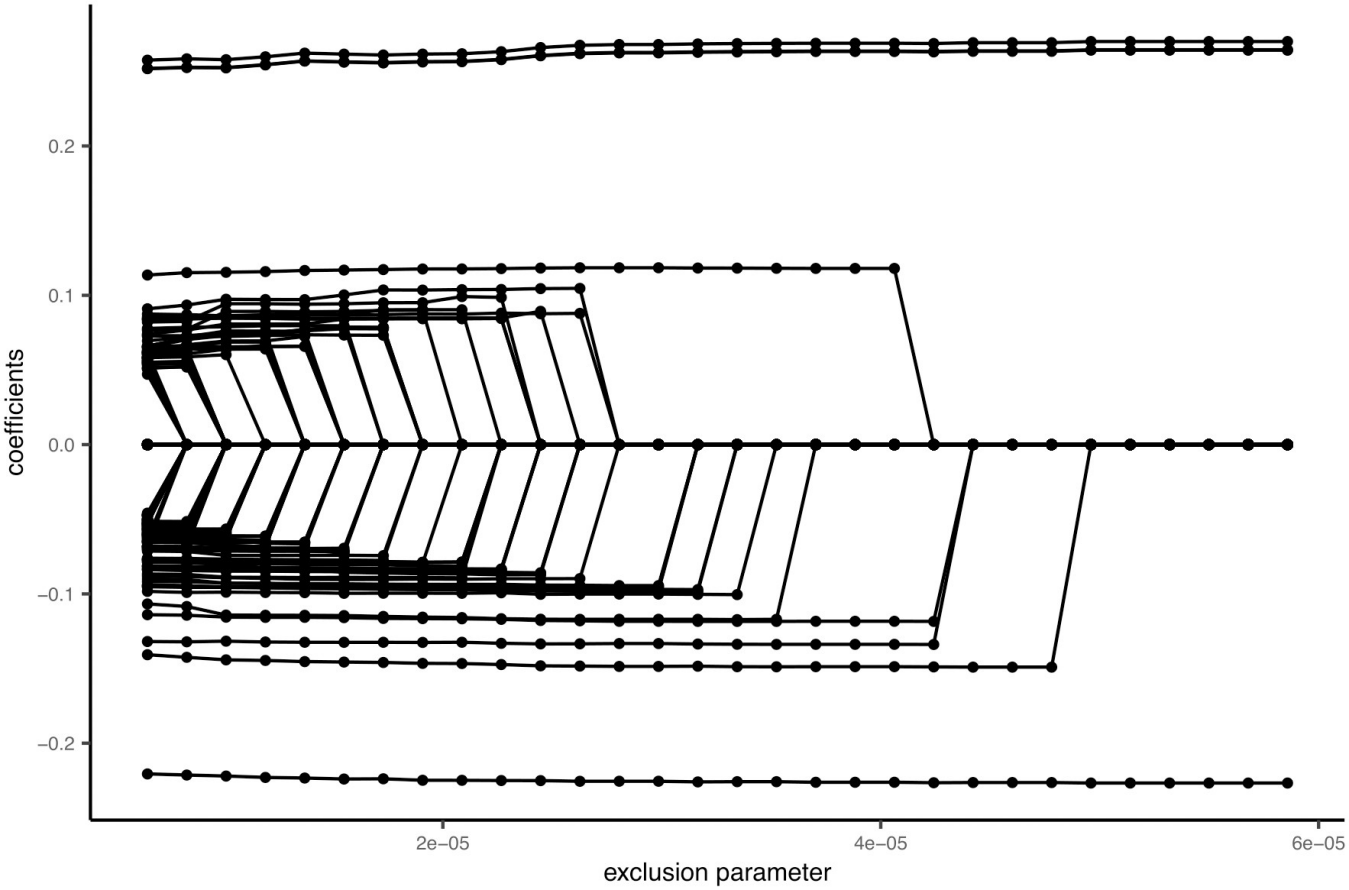
Regularization plot for rare variants based on the trio data from the Gabriella Miller Kids First Data Resource Center (DRC).

We applied TRIO_RVEMVS to a trio data set from the Gabriella Miller Kids First Pediatric Research Program (https://commonfund.nih.gov/kidsfirst/overview). Access to the data and analysis was exempted by the UTHealth IRB under protocol HSC-SPH-18–1127. On November 11, 2019, we obtained a total of 380 trios afflicted with cleft lip with or without cleft palate from the Gabriella Miller Kids First Data Resource Center (DRC). All authors confirmed that we did not have access to any information that could identify individual participants during and after data collection. We applied TRIO_RVEMVS to analyze chromosome 8 sequencing data for association with the risk of orofacial clefts in the European population. Quality control was performed using PLINK [51] according to the guidance from [52], including sample and marker genotyping efficiency/call rate, Mendelian inconsistency, and Hardy-Weinberg equilibrium. Subsequently, SHAPEIT2 [53] was utilized to phase the genotypes and obtain haplotypes for each trio of individuals. For TRIO_RVEMVS testing, our focus was on the region around 8q24 where the SNPs have been identified to be associated with the risk of orofacial clefts in the previous literature [54]. First, we identified the LD blocks in Chromosome 8 using Big-LD [55]. The LD block covering the region previously associated with orofacial clefts consists of 10401 SNPs after omitting singletons, doubletons, and tripletons from our analysis across the 380 trios (1140 individuals). We used the same procedure as described above for the simulated data to determine the exclusion parameters of the priors, which incorporates the regularization plot, Fig 6. The final selected SNPs were shown in Table 5. In total, we identified 8 SNPs in q24.21 and q24.22 associated with the risk of orofacial clefts in the Kids First European population.

**Table 5:** Final selected SNPs in the trio data from the Gabriella Miller Kids First Data Resource Center (DRC).

dbSNP	Position ref	MAF	Locus	Coefficients
rs1474668949	128825584	0.01	q24.21	-0.12
rs7017665	128946138	0.29	q24.21	0.26
rs17242358	128952627	0.29	q24.21	0.26
rs55658222	128963890	0.29	q24.21	0.27
rs1472381856	129156395	0.07	q24.21	-0.23
–	129243536	0.04	–	-0.15
rs1192270083	129364943	0.02	q24.21	-0.13
rs78061696	130619334	0.03	q24.22	0.12

## Discussion

Although new sequencing technologies and statistical methods have accelerated genome-wide association studies, a large portion of the genetic variability associated with birth defects remains to be discovered [6, 56–58]. These missing inheritances may reside in rare variants. Most existing genetic association methods, such as SKAT [28], PedGene [37], and RV-TDT [38], aggregate the burden of risks of rare variants within a region to test for association between that region and diseases. These methods often experience reduced power when a large number of unassociated rare variants are present within the region pooled, or when the rare variants are antagonistic within the same region (i.e. some promote risk while some offer protection against the disease) [29]. Additionally, it is well known that trio data is more robust to population stratification, and trio methods are well suited to help identify the risk of birth defects stemming from genetic variation [6, 56–59]. We developed a statistical tool based on trio family data, TRIO_RVEMVS, that jointly models common and rare variants to identify the rare variants driving the association of a genetic region with the disease rather than simply assessing genetic regions. One of the advantages of the proposed method is that the common and rare variants are detected simultaneously. The selection of rare variants does not need to be restricted to the region where common variants have been detected previously. Additionally, TRIO_RVEMVS can be potentially applied in fine-mapping studies, particularly following genome-wide association studies (GWAS) that have identified broad regions associated with certain phenotypes or diseases.

Using simulated data, we assessed the performance of TRIO_RVEMVS by comparing its performance at the region level with PedGene and RV-TDT using a weighted average correct association metric. We also examined the average true positive rate (ATPR) and average false positive rates (AFPR) when identifying individual variants. TRIO_RVEMVS outperformed PedGene when common variants were included whereas both methods were competitive when considering only rare variants. In this study, we also confirmed the result that PedGene outperformed RV-TDT whether common variants were included or not at the region level [50]. For 500 datasets with 1,500 trios, the ATPR was 2.45% and AFPR was 0.07% when both common and rare were considered at the individual level; the ATPR was 0.94%, and AFPR was 0.07% when the rare variants were considered. For 500 data sets with 350 trios, the ATPR was 4.33% and AFPR was 0.13% with common variants; ATPR was 0.62% and AFPR was 0.08% without common variants at the individual level.

When applying TRIO_RVEMVS to real data from the Gabriella Miller Kids First Data Resource Center (DRC), it identified 8 SNPs in q24.21 and q24.22 that were associated with the risk of orofacial clefts in the Kids First European population. Three SNPs were previously reported as common variants in locus 8q24. SNP rs7017665 has been reported in literature [60] and is highly correlated with another generally reported SNP, rs987525, with LD (*r*^2^ = 0.847, *D*′ = 0.983) [54, 60–62]. SNP rs55658222 and rs17242358 have both been previously reported in the literature [63, 64], respectively. One SNP we identified, rs78061696 has not yet been identified in the literature as associated with orofacial clefts.

We admit that one limitation of the proposed method is modeling the haplotype data which needs the assumption of accurate phasing. Therefore, accurate phasing is crucial before applying the proposed TRIO_EVEMVS. Fortunately, many phasing methods and software have been developed and different methods can be applied in different situations to achieve better accuracy [65]. For example, given the trio data, one can apply MERLIN [66], BEAGLE [67], and SHAPE-IT2 [53] et al. These methods work well for trios and parent-offspring pairs. In this study, we applied SHAPE-IT2 for phasing the real-data analysis. However, comparing different phasing methods and exploring their effect on the downstream analysis is beyond the scope of this study. Incorporating the uncertainty of phasing in the TRIO_EVEMVS is considered future research. Some challenges remain for TRIO_RVEMVS to detect rare variants: 1) TRIO_RVEMVS may fail to detect associated rare variants if their MAF is too small (less than 0.0027), and this threshold varies by sample size. 2) If there are too few rare variants associated with a disease in a given region, TRIO_RVEMVS may only detect the region and not detect the individual variant. 3) TRIO_RVEMVS may falsely detect some variants due to high LD. Despite these challenges, TRIO_RVEMVS is pioneering in its ability to identify individual rare variants alongside gene regions. Extending TRIO_RVEMVS to genome-wide data is considered as future research.

## Supporting information

S1 File Supplemental materials. (PDF)
