# Supplementary material for: TRIO RVEMVS: A Bayesian framework for rare variant association analysis with expectation-maximization variable selection using family trio data
Source: PLoS One. 2024 Dec 4;19(12):e0314502. doi: 10.1371/journal.pone.0314502 (PMC11616829; doi:10.1371/journal.pone.0314502)
Supplement: S1 File — (PDF) [file pone.0314502.s001.pdf]

TRIO_RVEMVS: A Bayesian framework for rare variant association analysis with expectation-maximization variable selection using family trio data

Supporting Information

Supplemental optimization algorithm: Mini-Batch SDCA

Goal: Minimize $P(\omega) = \frac{1}{N}\sum_{n=1}^{N}\phi_n(\omega) + g(\omega)$ where $\omega \in R^p$, $\phi_n(\omega) = \log(1 + \sum_{i=1}^{3} e^{-X_{in}\omega})$, $g(\omega) = \frac{1}{2N}\omega' P\omega$. The conjugate function of $g(\omega)$ is $g(\alpha) = \frac{N}{2}\alpha' P^{-1}\alpha$.
Parameters: $\theta \in [0,1]$; mini-batch size m, mini-batches $\{I_1, \cdots, I_{N/m}\}$
Initialize: $\alpha_1^0 = \cdots = \alpha_n^0 = \bar{\alpha}^{(t)} = 0$, $\alpha_i, \bar{\alpha} \in R^p$; $\omega^{(0)} = 0$;
Iterate: for epoch = 1, 2, $\cdots$
$\bar{I}^{(t-1)} = \varnothing$
for t = *epoch*, $2 \cdot$ *epoch*, $\cdots$
$u^{(t-1)} = (1-\theta)\omega^{(t-1)} + \theta\nabla g^*(\bar{\alpha}^{(t-1)})$
Randomly pick one mini-batch I from $\{I_1, \cdots, I_{N/m}\} - \bar{I}^{(t-1)}$ and update the associated dual variables
$\bar{I}^{(t)} = \bar{I}^{(t-1)} \cup I$
$\alpha_i(t) = (1-\theta)\alpha_i^{t-1} - \theta\nabla\phi_i(u^{(t-1)})$ for $i \in I$
$\alpha_j^{(t)} = \alpha_j^{(t-1)}$ for $j \notin I$
$\bar{\alpha}^{(t)} = \bar{\alpha}^{(t-1)} + \frac{1}{N}\sum_{i\in I}(\alpha_i^{(t)} - \alpha_i^{(t-1)})$
$\omega^{(t)} = (1-\theta)\omega^{(t-1)} + \theta\nabla g^*(\bar{\alpha}^{(t)})$
$\bar{I}^{(t)} = \bar{I}^{(t-1)} \cup I$
if $\bar{I}^{(t)} = \{I_1, \cdots, I_{N/m}\}$
end
end

Supplemental M-step

In this section, we show the details of solving the closed-form solutions to maximize Q_2, Q_3, and Q_4 at M-step. According to the E-step, Q_2 function can be written as

$$Q_2\left[\pi_1|\beta^{(k)}, \pi_1^{(k)}\right] = \sum_{s=1}^{S} E_{\gamma_s|\cdot}\left[\gamma_s\right]\log\left[\frac{\pi_1}{1-\pi_1}\right] + (2S-1)\log(1-\pi_1), \quad (1)$$

where

$$p_s^* = E_{\gamma_s|\cdot}[\gamma_s] = P(\gamma_s = 1|\beta^{(k)}, \pi_1^{(k)}) = \frac{a_s}{a_s + b_s}, \tag{2}$$

$a_s = P(\beta^{(k)}|\gamma_s = 1)P(\gamma_s = 1|\pi_1^{(k)})$, $b_s = P(\beta_s^{(k)}|\gamma_s = 0)P(\gamma_s = 0|\pi_1^{(k)})$ and $P(\gamma_s = 1|\pi_1^{(k)}) = \pi_1^{(k)}$. The first derivative of Q_2 with respective to π_1 is shown as

$$\frac{\partial Q_2}{\partial \pi_1} = \frac{1}{\pi_1}\sum_{s=1}^{S} p_s^* + \frac{1}{1-\pi_1}\sum_{s=1}^{S} p_s^* - \frac{1}{1-\pi_1}(2S-1). \tag{3}$$

The second derivative of Q_2 with respective to π_1 is shown as

$$\begin{aligned}\frac{\partial^2 Q_2}{\partial \pi_1} &= -\frac{1}{\pi_1^2}\sum_{s=1}^{S} p_s^* + \frac{1}{(1-\pi_1)^2}\sum_{s=1}^{S} p_s^* - \frac{1}{(1-\pi_1)^2}(2S-1) \\ &= -\frac{1}{\pi_1^2}\sum_{s=1}^{S} p_s^* - \frac{1}{(1-\pi_1)^2}(2S - \sum_{s=1}^{S} p_s^* - 1)\end{aligned} \tag{4}$$

According to Eq (2), $0 \geq p_s^* \leq 1$, then $\sum_{s=1}^{S} p_s^* \leq S$. When $S \geq 1$, we have $2S - \sum_{s=1}^{S} p_s^* - 1 \geq 0$, and $\frac{\partial^2 Q_2}{\partial \pi_1} \leq 0$. Let $\frac{\partial Q_2}{\partial \pi_1} = 0$, we obtain

$$\pi_1^{(k+1)} = \frac{\sum_{s=1}^{S} p_s^*}{2S-1} \tag{5}$$

And $\pi_1^{(k+1)}$ makes Q_2 reach the maximum value due to $\frac{\partial^2 Q_2}{\partial \pi_1} \leq 0$. The closed-form solutions to maximize Q_3 and Q_4 can be similarly derived.

Supplemental simulation detail

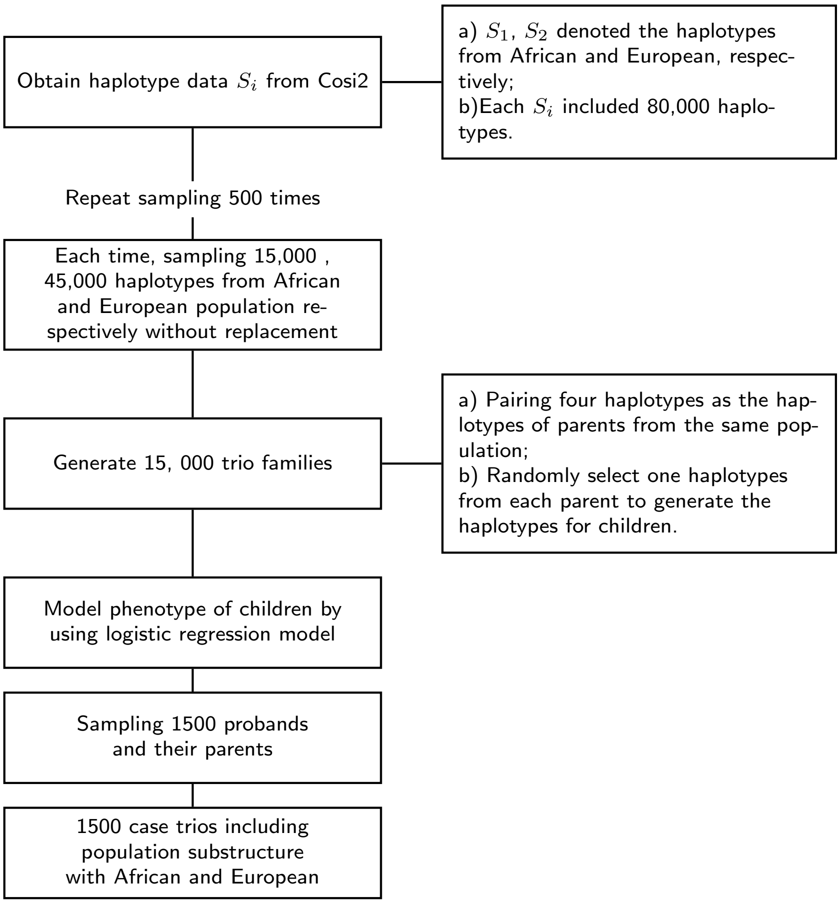

Fig S1. Simulation diagram.

Supplemental simulation exclusion parameters tuning

According to the parameter tuning rule in the section "Selection parameter tuning", for simulated data, we detail the parameter tuning procedure using the 1500 trio scenario (these details apply similarly to the smaller dataset scenario) as follows. We begin by setting the two exclusion parameters to be the same, i.e., $v_0 = v_2$. We use a regularization plot to identify a stable choice for the exclusion parameter of common variants, v_0. The range of possible values of v_0 to investigate with the regularization plot results in values that translate to a 95% confidence interval for the odds ratio ranging from [0.99, 1.01] to [0.97,1.03], Fig S2. According to the regularization plot, we choose the exclusion parameter such that the 95% prior probability for an odds ratio for excluded common variants is between [0.972, 1.028], $v_0 = 0.0002$. Once the value for the exclusion parameter for common variants was identified, $v_0 = 0.0002$, we repeated the process using regularization plots with respect to the exclusion parameter, v_2, considering values that generate 95% confidence intervals for the odds ratio to range from [0.995, 1.005] to [0.98, 1.02], Fig S3. It may be helpful to refine v_2

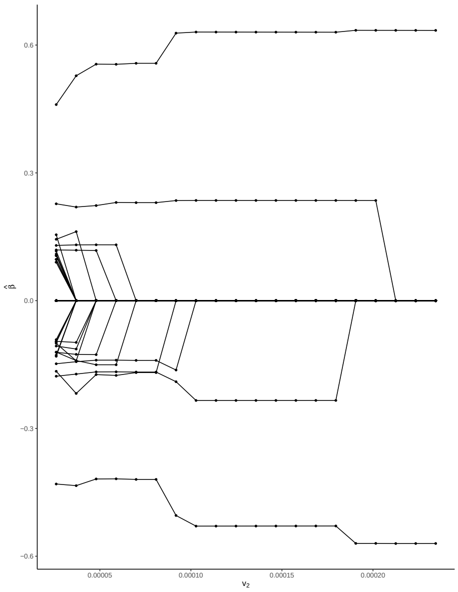

Fig S2. Regularization plot with respect to v_0 and v_2 ($v_0 = v_2$) at temperature $1/t$= 10. The range of v_0 was from $v_0 = 2.6 \times 10^-5$, corresponding to a 95% probability interval for an odds ratio of an excluded SNP to be [0.99,1.01], to $v_0 = 0.00023$, corresponding to a 95% probability interval for an odds ratio being [0.97,1.03].

by repeating the regularization plot across a finer grid consisting of the first 10 points of Fig S3 (see Fig S4). We chose the value of the exclusion parameter v_2 for rare variants in the following way: We require the corresponding 95% probability interval for the odds ratio to fall in the range of [0.995, 1.005] to [0.985, 1.015], which translate to a range of v_2 from 6.51×10^{-6} to 5.28×10^{-5}. We pick the value of v_2 such that there is no shrinkage in the regularization plot for three consecutive values of v_2, and we choose the 3rd point as the value for our exclusion parameter of v_2. Therefore, the exclusion parameter of rare variants for this data set was set as the 29th point in the regularization plot, i.e. $v_0 = 5.7 \times 10^{-5}$, corresponding to a 95% probability interval for the odds ratio being [0.985,1.015]. Based on the tuned exclusion parameters, we specified the region and individual variable selection in each data set.

Supplemental simulation results

In this section, we discuss the individual-level variant selection performance of TRIO_RVEMVS when all variants were considered, i.e., variants that were not polymorphic across all datasets. Considering that most of the rare variants were not polymorphic across all data sets, we defined the Average True and

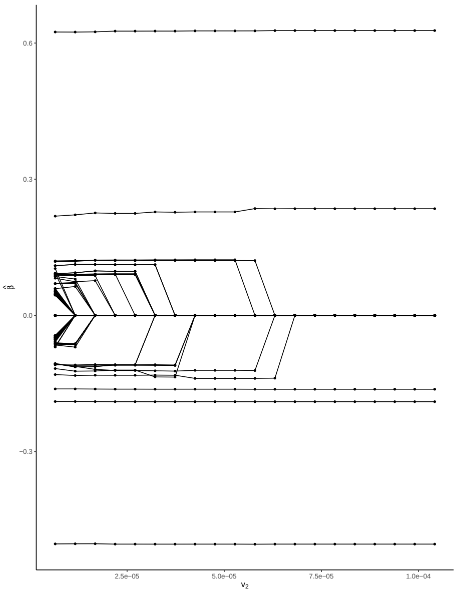

Fig S3. Regularization plot with respect to v_2 given $v_0 = 0.0002$, at temperature $1/t$= 10. The range of v_2 was from $v_2 = 6.51 \times 10^{-}6$, which corresponds to a 95% probability interval for an excluded SNP to range from [0.995,1.005], to $v_2 = 0.0001$, which corresponds to a 95% probability interval for odds ratio of an excluded SNP to be [0.98,1.02].

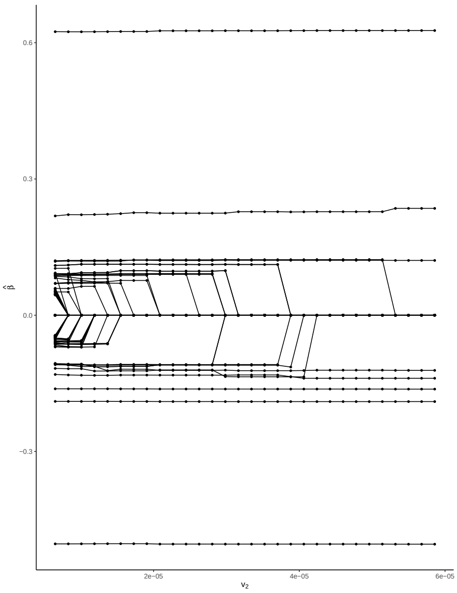

Fig S4. Regularization plot that zoomed in range of the first 10 points of v_2 in Fig S3.

False Positive Rate (ATPR and AFPR) for individual variants as follows:

$$\begin{aligned}
\text{ATPR} &= \frac{1}{\#\text{ of data sets}} \sum_{\text{data set } d} \frac{N_d(\text{selected}|\text{associated})}{N_d(\#\text{ of polymorphic associated variants})} \\
\text{AFPR} &= \frac{1}{\#\text{ of data sets}} \sum_{\text{data set } d} \frac{N_d(\text{selected}|\text{unassociated})}{N_d(\#\text{ of polymorphic unassociated variants})}
\end{aligned} \tag{6}$$

where $N_d(\text{selected}|\cdot)$ denotes the number of detected variants given the variants are associated or unassociated in data set d.

When both common and rare variants were considered, the ATPR, Eq (6), was 2.45%, and the AFPR was 0.07%; when only rare variants were considered the ATPR was 0.94% and AFPR was 0.07%. The ATPR and AFPR across varying MAF ranges are summarized in Table S1. Among the data sets with 1500 case-trios, the average number of polymorphic associated rare variants was 130, and the average number of associated singletons was 105 (about 80% of polymorphic causal rare variants were singletons). Excluding singletons, the ATPR was 11.90%, and AFPR was 0.28% when both common and rare variants were included in the analysis; when only considering rare variants, the ATPR was 4.87% and AFPR was 0.48%.

Table S1. The average true and false positive rate of individual variants detection in different MAF ranges.

	Sample size	MAF<0.01	$0.01 \leq$ MAF<0.05	MAF$\geq$ 0.05	0<MAF<0.5
ATPR (%)	1500	0.22	94.2	100	2.45
	350	0	25.37	83.8	4.33
AFPR (%)	1500	0	6.02	0.07	0.07
	350	0	2.62	1.39	0.13

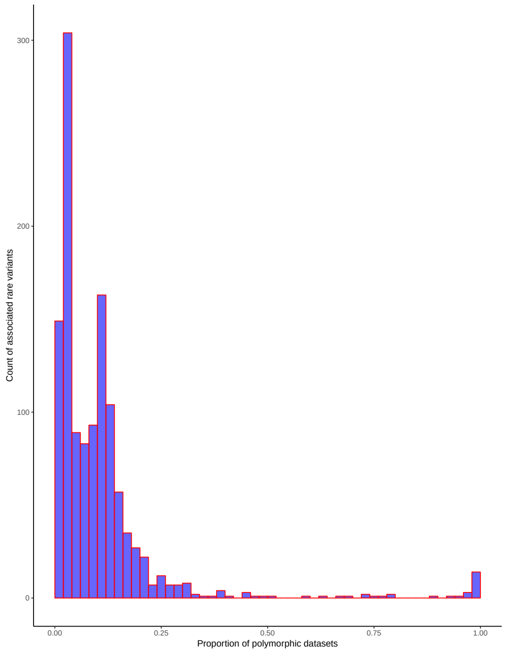

Fig S5. In the simulated 500 trio data sets, the histogram for the proportion of data sets in which associated rare variants are polymorphic.
